# Integrating SIF and Clearness Index to Improve Maize GPP Estimation Using Continuous Tower-Based Observations

**DOI:** 10.3390/s20092493

**Published:** 2020-04-28

**Authors:** Jidai Chen, Xinjie Liu, Shanshan Du, Yan Ma, Liangyun Liu

**Affiliations:** 1 Key Laboratory of Digital Earth Science, Aerospace Information Research Institute, Chinese Academy of Sciences, Beijing 100094, China; chenjidai@aircas.ac.cn (J.C.); duss@radi.ac.cn (S.D.); mayan2017@radi.ac.cn (Y.M.); liuly@radi.ac.cn (L.L.); 2 University of Chinese Academy of Sciences, Beijing 100049, China

**Keywords:** solar-induced chlorophyll fluorescence (SIF), gross primary production (GPP), clearness index (CI), tower-based observation, automatic observation system, eddy covariance flux, remote sensing

## Abstract

Solar-induced chlorophyll fluorescence (SIF) has been proven to be well correlated with vegetation photosynthesis. Although multiple studies have found that SIF demonstrates a strong correlation with gross primary production (GPP), SIF-based GPP estimation at different temporal scales has not been well explored. In this study, we aimed to investigate the quality of GPP estimates produced using the far-red SIF retrieved at 760 nm (SIF_760_) based on continuous tower-based observations of a maize field made during 2017 and 2018, and to explore the responses of GPP and SIF to different meteorological conditions, such as the amount of photosynthetically active radiation (PAR), the clearness index (CI, representing the weather condition), the air temperature (AT), and the vapor pressure deficit (VPD). Firstly, our results showed that the SIF_760_ tracked GPP well at both diurnal and seasonal scales, and that SIF_760_ was more linearly correlated to PAR than GPP was. Therefore, the SIF_760_–GPP relationship was clearly a hyperbolic relationship. For instantaneous observations made within a period of half an hour, the R^2^ value was 0.66 in 2017 and 2018. Based on daily mean observations, the R^2^ value was 0.82 and 0.76 in 2017 and 2018, respectively. Secondly, it was found that the SIF_760_–GPP relationship varied with the environmental conditions, with the CI being the dominant factor. At both diurnal and seasonal scales, the ratio of GPP to SIF_760_ decreased noticeably as the CI increased. Finally, the SIF_760_-based GPP models with and without the inclusion of CI were trained using 70% of daily observations from 2017 and 2018 and the models were validated using the remaining 30% of the dataset. For both linear and non-linear models, the inclusion of the CI greatly improved the SIF_760_-based GPP estimates based on daily mean observations: the value of R^2^ increased from 0.71 to 0.82 for the linear model and from 0.82 to 0.87 for the non-linear model. The validation results confirmed that the SIF_760_-based GPP estimation was improved greatly by including the CI, giving a higher R^2^ and a lower RMSE. These values improved from R^2^ = 0.66 and RMSE = 7.02 mw/m^2^/nm/sr to R^2^ = 0.76 and RMSE = 6.36 mw/m^2^/nm/sr for the linear model, and from R^2^ = 0.71 and RMSE = 4.76 mw/m^2^/nm/sr to R^2^ = 0.78 and RMSE = 3.50 mw/m^2^/nm/sr for the non-linear model. Therefore, our results demonstrated that SIF_760_ is a reliable proxy for GPP and that SIF_760_-based GPP estimation can be greatly improved by integrating the CI with SIF_760_. These findings will be useful in the remote sensing of vegetation GPP using satellite, airborne, and tower-based SIF data because the CI is usually an easily accessible meteorological variable.

## 1. Introduction 

Photosynthesis, the most important biochemical process in terrestrial ecosystems, is an essential part of the global carbon cycle [1,2]. Therefore, as an indicator of photosynthetic carbon exchange in ecosystems, accurate observations of the gross primary production (GPP) and a clear explanation of its response to environmental conditions are required, in order to help solve the problem of climate change [3,4,5].

The eddy covariance (EC) technique is a terrestrial observation method used to estimate the GPP, and the continuously measured data are relatively accurate [6]. However, an EC tower only has a small field of view and limited aerodynamic properties [7], which makes it difficult to measure GPP at larger scales using this method. At larger spatial scales, a measured approach using models and algorithms that integrate tower-based measurements with remotely sensed data offers the possibility to estimate GPP [8,9]. Monteith [10] proposed the light-use efficiency (LUE) based model, validated using tower-based data from an EC tower, which is well-known for its simplicity and efficiency and has been widely applied for remote sensing estimates of GPP. LUE model approaches are commonly based on reflectance vegetation indices [11], such as the normalized difference vegetation index (NDVI) and the enhanced vegetation index (EVI) [12,13]. However, the NDVI and EVI are related to changes in the canopy structure and biomass, and, therefore, it is hard to capture the diurnal and seasonal dynamics of vegetation photosynthesis in this way [14]. As a result, estimating the GPP using these traditional remote sensing methods remains a challenge, and there is a critical need to develop a more accurate method to estimate GPP and characterize its temporal variations for different vegetation types and environmental conditions [7,15,16].

During photosynthesis, green components absorb sunlight covering the 400–700 nm range of the electromagnetic spectrum, triggering chlorophyll to emit the red and far-red light known as solar-induced chlorophyll fluorescence (SIF). Numerous studies have demonstrated a strong relationship between GPP and SIF based on satellite, airborne, tower-based and ground platforms [2,6,7,9,16,17,18,19,20,21,22,23,24,25,26]. Airborne and satellite remote sensing can only offer a snapshot of the SIF at a certain time (for example, 13:30 local time in the case of the OCO-2 satellite), while the influence of the temporal scale used is difficult to assess using instantaneous observations [27,28,29]. Automated, continuous, tower-based SIF observation systems have been developed to explore the dynamic mechanism between SIF and GPP [25,26]. Tower-based SIF studies are continuing to collect long-term data sets for different ecosystems, including wheat [29], maize [30], rice paddy [31], soybean [32], sorghum [33], shrub [34], temperate forest [35], deciduous forest [26], and evergreen forest [36]. These observations provide direct and reliable evidence that can be used to explore the correlation between SIF and GPP at different temporal scales.

Although multiple studies have found that SIF demonstrates a strong linear relationship with gross primary production (GPP), the SIF–GPP relationship at different temporal scales and under different environmental conditions has not been well explored. Some studies have found that the relationship has a weaker empirical linear correlation over shorter time scales than at the seasonal scale [29,37]. Van der Tol et al. [38] demonstrated that the relationship between SIF and GPP can be nonlinear over short temporal scales. Zarco-Tejada et al. [39] assessed the relationship between airborne-measured SIF and field-measured leaf CO_2_ assimilation in a citrus crop field, and, using second-order polynomial regression, found that the SIF and leaf carbon assimilation exhibited a statistically significant relationship at the diurnal scale. However, Damm et al. [6] showed that the relationship between canopy SIF and GPP was asymptotic and scaled with environmental variables and the temporal resolution for crop fields. Although much understanding of the response of GPP to SIF has been gained from numerous SIF-based GPP models at the leaf and canopy scales [14,32,40,41,42], there is general agreement among these models that the accuracy of GPP estimates is affected by the light conditions and environmental variables. In addition, different models may have different sensitivities to the timescales and environmental variables used for predicting canopy photosynthesis production.

Generally, CI has been used as an indicator of solar light conditions that can distinguish different weather conditions, such as sunny days and cloudy days [30,43]. Several studies have demonstrated that weather conditions can influence the canopy GPP and SIF. For example, Smith et al. [44] observed that an understory herb in a mixed spruce stand had a stronger ability to gain carbon on representative cloudy days than clear days. Gu et al. [45] found that the effect of different environmental factors on ecosystem carbon sequestration was complex, especially under cloudy conditions. Diffuse and direct radiation differed in the way they were transmitted though the canopy, and the diffuse radiation had a much lower tendency to cause canopy photosynthetic saturation in the case of forest, tall grass, and winter wheat ecosystems [46]. Campbell et al. [40] also showed that the relationship between GPP and SIF differed depending on the light conditions. Previous studies have demonstrated that CI is usually related to other environmental parameters, such as the temperature, humidity, and precipitation. Recently, Yang et al. [31] found that the ratio of GPP to SIF generally increased along with the relative humidity (RH) and confirmed that incorporating the relative humidity and growth stage into multiple regression analyses led to improvements in estimates of GPP for a rice paddy. However, irrespective of whether a linear model or non-linear model is used for SIF-based GPP estimation, the effects of CI are still unknown, which is important for an accurate assessment of GPP. On the other hand, although current studies have found the absorbed photosynthetically active radiation (APAR) is the dominant factor in the SIF–GPP relationship, Yang et al. [35] recently noted that SIF–GPP relationships were not only driven by APAR, but were also strongly correlated with the leaf-level biochemistry and canopy structure at diurnal and seasonal scales in temperate deciduous forests. Therefore, future studies should pay more attention to the effects of the plant physiology, canopy structure, and environmental conditions on the correlation between SIF and GPP across the growing season. Overall, the strong link between SIF and photosynthesis has permitted the development of a new approach for accurately estimating GPP at different spatial and temporal scales [47].

In this study, we deployed an automated tower-based SIF system in a maize field to collect continuous observations of far-red SIF, which were then paired with measurements made at an existing EC flux tower so that carbon exchange data were collected simultaneously. The acquired data covered the growing seasons of 2017 and 2018. The objectives were: (1) to investigate the ability of far-red SIF to track maize GPP at diurnal and seasonal scales, (2) to explore the impact of the environmental conditions on the SIF–GPP relationship, and (3) to improve the estimation of GPP by integrating SIF measurements and the clearness index.

## 2. Materials and Methods

### 2.1. Site Description

**Figure 1:**
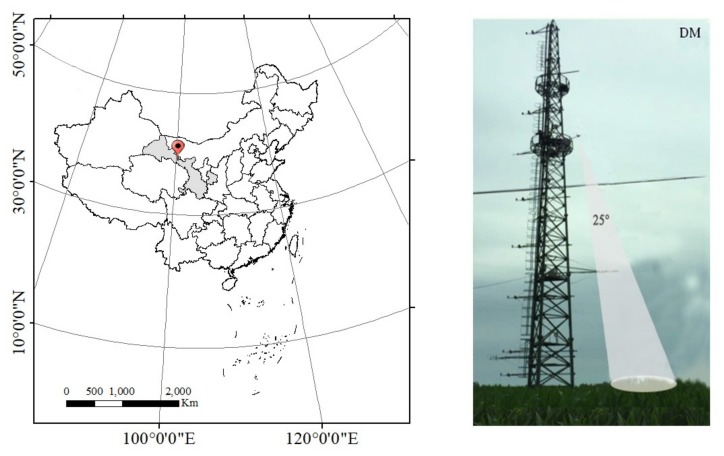
Location of the study site and observation tower.

The DaMan (referred to as DM hereafter) site is located in the DaMan irrigation district in the middle reaches of the Heihe River Basin, which is a typical oasis and lies on flat terrain approximately 8 km southeast of Zhangye, Gansu Province, in northwest China (38°51′20″N, 100°22′20″E)(the location is shown in Figure 1). This region has a typical temperate continental climate, with an average annual temperature of 8 ℃, annual sunshine hours of 3000–3600 h, and annual precipitation of about 300 mm. The dominant crop type in this region is single-cropping maize with no special irrigation and fertilization controls [48,49].

The spectral measurements at the DM site was made from 8 June 2017 to 30 September 2017, and 7 June 2018 to 10 September 2018. In 2017, some days’ data was missing (12 to 22 July, and 22 August to 10 September) due to hardware failure. In total, 189 days of measurements were obtained at the DM site during the two growing seasons of 2017 and 2018.

### 2.2. Measurements of CO_2_ Fluxes and Meteorological Variables

A CO_2_ flux observation system based on the eddy covariance (EC) technique [50,51], was used to measure the CO_2_ exchange between the maize canopy and the atmosphere. The EC system was fixed to a 5-m high platform on a flux tower located in the DM irrigation district. The turbulent flux data were sampled at a frequency of 10 Hz [27] and stored by a data logger (CR5000 at YK and GT, Campbell Scientific Inc., Logan, UT, USA). The raw EC data were processed to obtain the net ecosystem exchange of CO_2_ (NEE). Baldocchi et al. [52] have reported that the in situ GPP can be estimated from the EC-measured NEE and ecosystem respiration (Re) using the equation GPP = R2 − NEE. In addition, Liu et al. [53] found that the EC-measured net ecosystem exchange (NEE) was equivalent to the night-time ecosystem respiration. Goulden et al. [54] also reported that the night-time Re was related to the air temperature relationship. Therefore, we used the night-time partitioning algorithm to partition the GPP (as the measured values of GPP) from the daytime NEE values in this study. In addition, half-hourly mean and daily mean data were processed using the averaging method proposed in Hu et al. [28].

Environmental and meteorological variables, including the photosynthetically active radiation (PAR), air temperature (AT), and humidity (RH), were continuously observed using an automatic weather station (AWS) system, which was also fixed to the EC flux tower. The AWS included a 3D sonic anemometer (CSAT3, Campbell Scientific Inc., Logan, UT, USA) and an open-path infrared gas analyzer (Li-7500, Li-Cor, Lincoln, NE, USA). PAR and all other meteorological variables were averaged to 30-min values to correspond to the flux observations.

The vapor pressure deficit (VPD) was quantified as follows [55]:

(1) $$\mathrm{VPD}=0.611\times e^{\frac{17.27\times \mathrm{AT}}{\mathrm{AT}}}\times \left(1-\frac{\mathrm{RH}}{100}\right),$$

where AT is the air temperature and, RH indicates the relative humidity.

For a better understanding of the effects of different incident light conditions on the SIF–GPP relationship, we considered the overall influence of the solar zenith angle (SZA) and PAR.

The clearness index (CI) is an important weather metric that is used to describe the relative intensity of solar incident radiation. In this study, CI was quantified as the ratio of the solar radiation arriving at the top of canopy to the solar radiation at the top of atmosphere. Therefore, half-hourly CI (${\mathrm{CI}}_{30}$) was calculated using the following formula [56]:

(2) $${\mathrm{PAR}}_{30}=S_{0}\times \left(1+0.033\times \cos\left(2\pi \times \mathrm{DOY}/365\right)\right)\times \cos\left({\mathrm{SZA}}_{T30}\right),$$

(3) $${\mathrm{CI}}_{30}={\mathrm{PAR}}_{T30}/{\mathrm{PAR}}_{30},$$

where ${\mathrm{PAR}}_{T30}$ represents the magnitude of PAR over a 30-min interval, ${\mathrm{PAR}}_{30}$ represents the maximal PAR at the top of atmosphere (TOA), also over a 30-min interval, and $S_{0}$ is the solar constant (1367 W/m^2^).

The daily CI (${\mathrm{CI}}_{\mathrm{day}}$) was calculated as follows:

(4) $$\mathrm{PAR}=\int_{T_{sunrise}}^{T_{sunset}}\mathrm{PAR}\left(t\right)dt\approx \sum_{T_{sunrise}}^{T_{sunset}}{\mathrm{PAR}}_{T30}\left(t\right)\times \Delta t_{30},$$

(5) $${\mathrm{CI}}_{\mathrm{day}}={\mathrm{PAR}}_{\mathrm{day}}/{\mathrm{PAR}}_{0},$$

where $\Delta t_{30}$ is the 30-min temporal interval, ${\mathrm{PAR}}_{\mathrm{day}}$ is the integrated value of all half-hourly PAR measurements made from sunrise to sunset in Equation (4), and ${\mathrm{PAR}}_{0}$ represents the sum of daily TOA PAR [56].

### 2.3. Measurements of Solar-Induced Fluorescence

**Figure 2:**
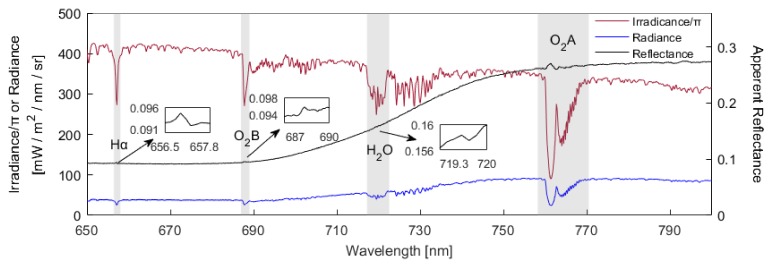
A sample of the irradiance, radiance, and apparent reflectance measured by the automatic observation system at about 13:30 on June 15, 2017 at the DaMan (DM) site. The four gray shaded regions indicate the Hα absorption centered around 656 nm, the O_2_-B absorption at around 688 nm, the water vapor absorption band at around 720 nm, and the O_2_-A absorption region around 760 nm. The O_2_-A absorption band, which more clearly strengthened the weak reflected signal, was used to extract the canopy solar-induced chlorophyll fluorescence (SIF) of maize.

An automatic long-term SIF observation system was fixed to an 18-m high platform on the flux tower at the DM site (Figure 1). The SIF observation system mainly consisted of a customized Ocean Optics QE65PRO spectrometer (Ocean Optics, Dunedin, FL, USA), an automatic refrigeration system, and a control PC for data collection and storage. The Ocean Optics QE65PRO spectrometer covers 645–805 wavelength ranges with a high spectral resolution of around 0.34 nm, a sampling interval of about 0.17 nm, and a signal to noise ratio (SNR) higher than 1000 [27]. The spectrometer splits the optical signal into two channels to measure both downwelling incident radiance and upwelling radiance simultaneously using a cosine corrector (CC3-3-UV-S, Ocean Optics, Inc., Dunedin, FL, USA) and a conical fore-optic (bare fiber), respectively. In the upward direction, the cosine corrector can capture the downwelling incident radiance with a large field view (FOV) of 180°, and the conical fore-optic can capture the upward radiance with a small FOV of about 20° when pointing at the maize canopy. The measuring mode was set to a ‘sandwich’ type: that is, by alternately opening the up and down channels, the downwelling solar irradiance was first collected, the upwelling irradiance of the canopy was then measured, and the downwelling solar irradiance was finally collected again (Figure 2). To reduce the effects of weather changes caused by the time delay, we calculated the average of the two downwelling solar irradiance measurements. Generally, it takes about 15 s at midday for the observation system to complete a spectral measurement cycle, and about 2 min at sunrise or sunset. Before each of the measurements, the integrated time was optimized depending on the light intensity, and it was generally 0.7–6 s.

SIF retrieval algorithms are generally based on the Fraunhofer Line Discrimination (FLD) principle. In this study, we applied the 3FLD approach to acquire the SIF signals, assuming that the fluorescence and reflectance varied linearly: this approach has been proven to be more robust and accurate than the improved FLD method [57,58]. The 3FLD method needs spectral data from three bands, including one absorption band and the two shoulders of the absorption band. Although some studies have reported that the red band SIF may contain more information about photosystem II (PS II), the canopy structure and varying solar-view geometries have more complex effects on the red SIF than on the far-red SIF [9,59]. Therefore, in order to better rule out the influence of retrievals and the strong re-absorption, we only considered the relationship between canopy GPP and far-red SIF.

For the O_2_-A band, we employed 761 nm as the inside channel, and 755 nm and 771 nm were taken as the shorter- and longer-wavelength channels, respectively. The weights for the two shoulders of the absorption band can be expressed as

(6) $$w_{755}=\frac{\rho_{771}-\rho_{761}}{\rho_{771}-\rho_{755}}\wedge w_{771}=\frac{\rho_{761}-\rho_{755}}{\rho_{771}-\rho_{755}},$$

where w is the weight coefficient, ρ represents the reflectance, and the subscripts indicate the wavelengths adjacent to the absorption valley. Then, the SIF can be calculated using

(7) $$SIF=\frac{\left(E_{755}w_{755}+E_{771}w_{771}\right)L_{761}-E_{761}\left(L_{755}w_{755}+L_{771}w_{771}\right)}{\left(E_{755}w_{755}+E_{771}w_{771}\right)-E_{761}},$$

where E represents the solar irradiance, and L represents the canopy reflectance.

In addition, as the strong absorption effect at the O_2_-A absorption bands has an obvious influence on SIF retrieval, atmospheric correction is required even for tower-based SIF observations made with a sensor located tens of meters above the canopy [60]. Therefore, we established look-up tables (LUTs) to estimate the upward and downward transmittance using the aerosol optical depth (AOD) and the radiative transfer path length (RTPL) based on moderate resolution atmospheric transmission 5 (MODTRAN 5) model simulations and used the atmospheric transmittance to correct the SIF measurements.

### 2.4. Statistical Analysis of the SIF_760_–GPP Relationship

To avoid the impact of low solar illumination, measurements that were collected at a solar zenith angle (SZA) > 80° were excluded from the analysis. Raw SIF data that were negative or had values higher than 2 mW/m^2^/nm/sr were also not used in the subsequent data processing and analysis. In addition, outliers (measurements outside the range μ±3σ) were also excluded from the diurnal relationship analysis (μ and σ are the mean and standard deviation, respectively). Some studies have reported that the relationship between the canopy SIF and GPP exhibits a non-linear tendency and depends on the environmental variables [39], especially the light conditions. In this study, we evaluated the performance of canopy estimates based on the linear and non-linear regression model based on $SIF_{760}$ and explored the effects of the CI on the linear and non-linear relationship between $SIF_{760}$ and GPP. The linear and hyperbolic regression models characterizing the relationships between GPP and $SIF_{760}$ are [61]:

(8) $$GPP_{L}=f_{L}\left(SIF\right)=a\times SIF_{760},$$

(9) $$GPP_{NL}=f_{NL}\left(SIF\right)=a\times \frac{SIF_{760}}{SIF_{760}+b},$$

where $GPP_{L}$ is the GPP predicted by the linear model; $GPP_{NL}$ is the GPP predicted by the non-linear model; a and b are the fitting parameters [38]; and $f_{L}$ and $f_{NL}$ represent the linear regression model and the non-linear regression model, respectively.

Previous studies have reported that the weather conditions can influence GPP estimates made using the light-use efficiency model [62,63]. As CI influences the relationship between SIF and GPP, we added CI into the SIF-based GPP estimation models (both linear and non-linear), which are expressed as

(10) $${\mathrm{GPP}}_{L-\mathrm{CI}}=f_{L}\left(\mathrm{SIF}\right)\times g_{L}\left(\mathrm{CI}\right),$$

(11) $${\mathrm{GPP}}_{\mathrm{NL}-\mathrm{CI}}=f_{NL}\left(\mathrm{SIF}\right)\times g_{NL}\left(\mathrm{CI}\right),$$

where ${\mathrm{GPP}}_{L-\mathrm{CI}}$ is the GPP predicted by the linear model considering the effects of CI, ${\mathrm{GPP}}_{\mathrm{NL}-\mathrm{CI}}$ is the GPP predicted by the non-linear model considering the effects of CI, $g_{L}\left(\mathrm{CI}\right)$ describes the relationship between GPP/GPP_L_ and CI for the linear SIF_760_-based GPP model, and $g_{NL}\left(\mathrm{CI}\right)$ represents the relationship between GPP/GPP_NL_ and CI for the non-linear SIF_760_-based GPP model. Therefore, $g_{L}\left(\mathrm{CI}\right)$ or $g_{NL}\left(\mathrm{CI}\right)$ can be fitted using the ratio between the measured GPP (true values of GPP) and estimated GPP by $f_{L}\left(\mathrm{SIF}\right)$ or $f_{NL}\left(\mathrm{SIF}\right)$.

## 3. Results

### 3.1. Diurnal Dynamics of SIF and GPP at the Canopy Scale

**Figure 3:**
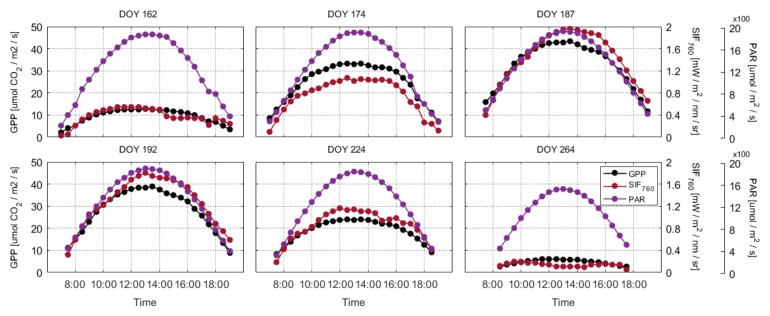
The diurnal variations in canopy gross primary production (GPP, black), far-red solar- induced fluorescence (SIF_760_, dark red), and photosynthetically active radiation (PAR, purple) under clear-sky conditions at the DM flux site in 2017. DOY represents the day of the year. The first row represents the vegetative stage (DOY 162, DOY 174, and DOY 187) and the second row indicates the reproductive stage (DOY 192, DOY 224, and DOY 264).

**Figure 4:**
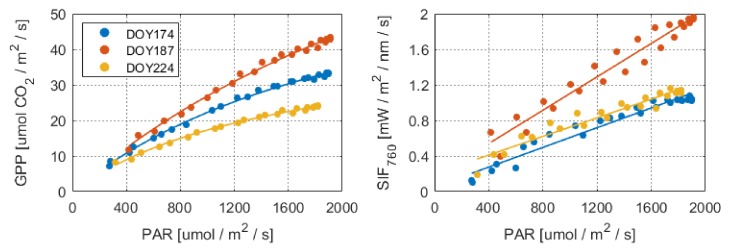
Relationships between PAR and canopy GPP and SIF_760_ based on individual day data acquired over the whole growing season at the DM maize field in 2017. The GPP–PAR relationships can be well fitted using a hyperbolic function, while the SIF_760_–PAR relationships can be well fitted using the linear regression method. The DOY indicates the day of the year.

We analyzed the dynamic variations in GPP, SIF, and PAR for the maize canopy on individual clear-sky days at the diurnal timescale over the whole growing season (Figure 3). The SIF and GPP showed a strong diurnal pattern with a steady increase during the morning and subsequent decrease after solar noon, which was mainly driven by the incoming radiation. Both the SIF and GPP exhibited gradual increases with PAR at the diurnal timescale (Figure 4). The diurnal relationships between GPP and PAR exhibited a clear asymptotic trend, while the SIF–PAR relationships showed a more linear trend. In addition, the structural and physiological variables had a significant effect on the saturation of GPP over the whole growing season; however, this effect was not considered in this study. Overall, the relationships between the canopy SIF_760_ and GPP were asymptotic at the diurnal scale, and PAR was the key factor driving the relationship between the SIF and GPP.

### 3.2. Seasonal Dynamics of SIF and GPP at the Canopy Scale

**Figure 5:**
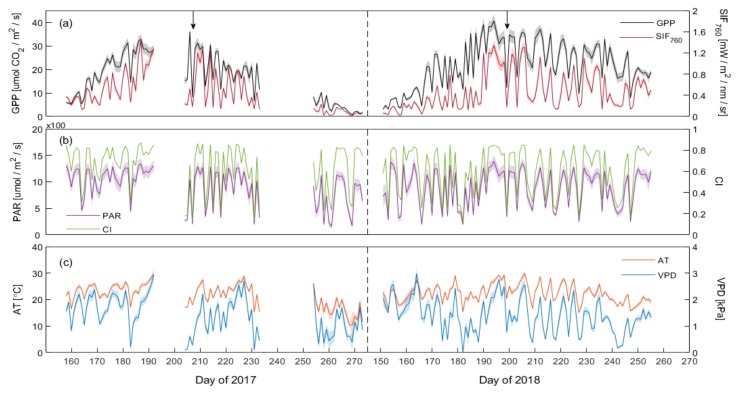
(**a**) Seasonal dynamics of canopy gross primary production (GPP, black) and solar induced fluorescence in the far-red band ($SIF_{760}$, dark red) in the DM maize field in 2017 and 2018. Shaded regions indicate values within ±SE of the mean; (**b**) photosynthetically active radiation (PAR, purple) and clear sky index (CI, green); (**c**) air temperature (AT, orange) and vapor pressure deficit (VPD, blue). The arrows indicate the decrease in GPP on individual days. All maize canopy measurements represent the daily mean values during each daily sampling period.

Figure 5 shows the processed data for two consecutive years at the DM observation site including the seasonal variations in SIF_760_, GPP, PAR, CI, AT, and VPD for the maize canopy. The seasonal pattern of SIF_760_ was consistent with that of the GPP over the whole growing season in 2017 and 2018 (Figure 5a). Both SIF_760_ and GPP exhibited gradual increases as the canopy developed and showed a significant decline from leaf senescence to harvest. In addition, SIF_760_ and GPP generally remained relatively stable with the exception of some cloudy sky days (Figure 5b). This can be seen from the decrease in GPP around DOY 206 to DOY 207 in 2017, and DOY 198 to DOY 199 in 2018 (marked with arrows in Figure 5a), for example. Therefore, we concluded that the seasonal fluctuations in SIF_760_ and GPP were also primary driven by the variations in PAR; this explained most of the changes in SIF_760_ and GPP. Similar seasonal trajectories were observed for both the clearness index (CI) and PAR over the whole growing season (Figure 5b). Therefore, the value of CI can be used as a proxy for the light conditions. In addition, AT and VPD presented similar seasonal patterns and their relationships with the ratio of GPP to SIF_760_ were relatively weak (Table 1).

**Figure 6:**
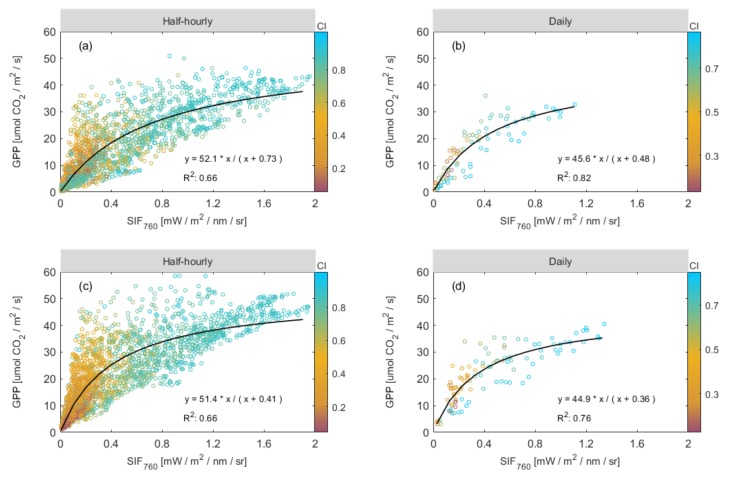
Relationships between canopy GPP and SIF based on half-hourly (**a**,**c**) and daily (**b**,**d**) mean data observed over the whole growing season in the DM maize field in 2017 (**a**,**b**) and 2018 (**c**,**d**). The color scale represents the value of the clearness index (CI). Hyperbolic regression lines are shown in black and the coefficient of determination (R^2^) for each best-fitting line is given.

Next, we analyzed the relationships between SIF_760_ and GPP based on the half-hourly and daily mean data for the whole growing season. At the seasonal timescale, the relationships between maize canopy GPP and SIF_760_ exhibited strong hyperbolic correlations at both half-hourly (R^2^ = 0.66, Figure 6a; R^2^ = 0.66, Figure 6c) and daily (R^2^ = 0.82, Figure 6b; R^2^ = 0.76, Figure 6d) temporal resolutions. Additionally, we found that the relationships between SIF_760_ and GPP showed an improvement when the half-hourly mean data were aggregated to daily mean data (R^2^ increased from 0.66 to 0.82, Figure 6a,b; R^2^ increased from 0.66 to 0.76, Figure 6c,d) due to the reduction in noise.

### 3.3. Improving the Estimation of GPP Using a Combination of SIF and CI

To further analyze the relationships between both SIF_760_ and GPP and relevant environmental variables across the growing period, we applied linear correlation analysis to investigate the effect of CI, AT, and VPD on SIF_760_ and GPP at different time scales (Table 1). Compared to other environmental variables, it was found that CI had a more significant effect on the variations in GPP and SIF_760_ at different time scales in 2017 and 2018. Therefore, it may be that CI is the dominant environmental variable in terms of the variations in the SIF_760_–GPP relationship. Furthermore, over the whole growing season, we found that GPP will saturate or increase more slowly when the CI is high (Figure 6). Maize canopy GPP exhibited a greater tendency to saturate under high levels of direct light but the saturation was less likely to occur when the amount of scattered light was extremely high. Therefore, it was decided that CI should be included in the SIF_760_-based GPP estimation model.

**Figure 7:**
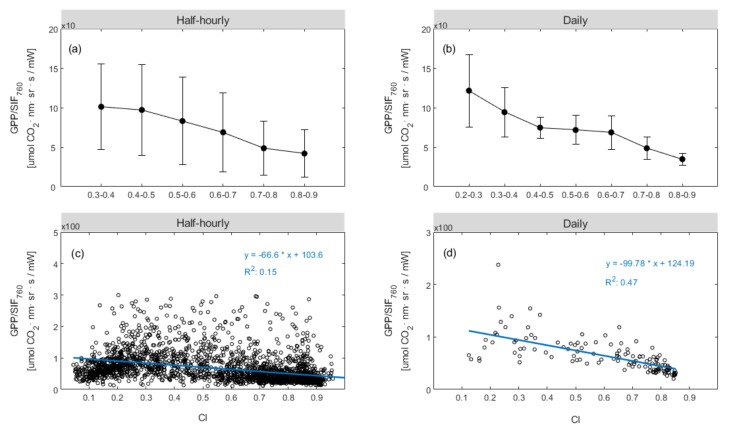
Dependence of canopy GPP/SIF_760_ on the clearness index (CI) averaged at 0.1 intervals (**a**,**b**) in the DM maize field for the training dataset. Half-hourly data with CI < 0.3 and daily data with CI < 0.2 were excluded from the analysis. The linear correlations between CI and GPP/SIF_760_ are based on the half-hourly mean data (**c**) and daily mean data (**d**), respectively. Error bars indicate ±SD from the mean.

Next, we investigated the influence of CI on the relationship between GPP and SIF_760_ based on half-hourly and daily measurements of the training dataset made in the DM maize field. Figure 7 illustrates the variation in the ratio of canopy GPP to SIF_760_ with changes in CI based on the training data. The ratio of GPP to SIF_760_ exhibits an obvious decreasing trend as CI increases (Figure 7a,b). For the half-hourly data, the correlation between CI and the ratio of GPP to SIF_760_ exhibits a weak relationship, with an R^2^ value of 0.17 (Figure 7c). However, the relationship between the ratio of GPP to SIF_760_ and CI shows a stronger correlation for the daily data, giving an R^2^ value of 0.51 (Figure 7d). Overall, these results show that the ratio of GPP to SIF_760_ decreases as CI increases, reflecting the differences in the response of GPP and SIF_760_ to the weather conditions.

From Figure 6, we found that under the same SIF value, when the CI value was small (indicated by a yellow circle), the corresponding GPP was large. Moreover, we can see from Figure 7 that the ratio of GPP to SIF_760_ exhibits an obvious decreasing trend as CI increases, reflecting the difference in the response of GPP and SIF_760_ to weather conditions. These results indicated that the CI was a key factor that affected the slope of GPP against SIF_760_ and the influence of CI should be introduced in the SIF760-based GPP estimation model. Therefore, we added CI to the SIF_760_-based GPP estimation models (both linear and non-linear), which can be seen in the Equations (10) and (11).

The relationship between the measured GPP (true values of GPP measured by the EC technique, referred to as $GPP_{meas}$ hereafter) and the predicted GPP based on SIF was used to establish a relationship with CI for both linear and non-linear SIF-based GPP estimation models. The ratio of $GPP_{meas}$ to the predicted GPP ($GPP_{L}$ or $GPP_{NL}$) represents the difference between the real GPP and GPP predicted by SIF for a linear or non-linear model. We found that this difference is related to CI for the linear SIF-based GPP estimation model and the $GPP_{meas}$/$GPP_{L}$ had a strong relationship with CI, as we can see in Figure 7, and we used this ratio to fit the relationship with CI and integrated it into the SIF_760_-based GPP model to improve the estimation accuracy of GPP. By comparing the results of the linear model with and without CI, it was shown that CI is indeed an important factor that affects the slope of GPP against SIF_760_ (Table 2).

**Figure 8:**
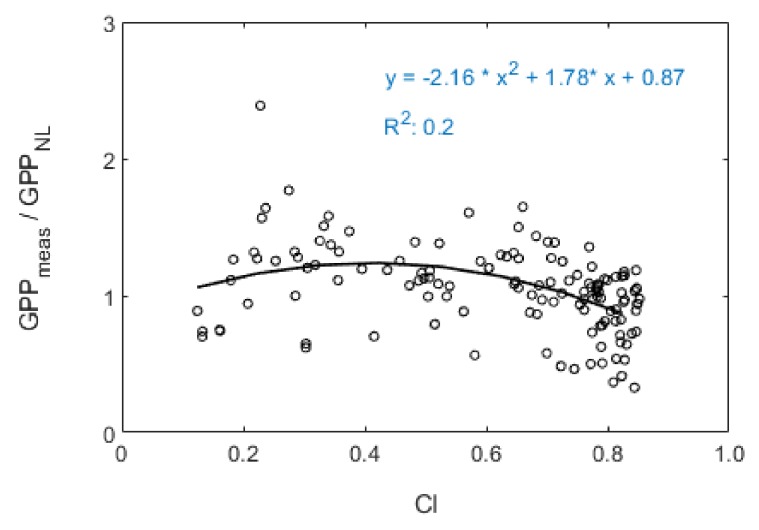
Relationship between CI and the ratio of measured canopy GPP ($GPP_{meas}$) to predicted GPP by the non-linear SIF_760_-based GPP estimation model ($GPP_{NL}$).

**Figure 9:**
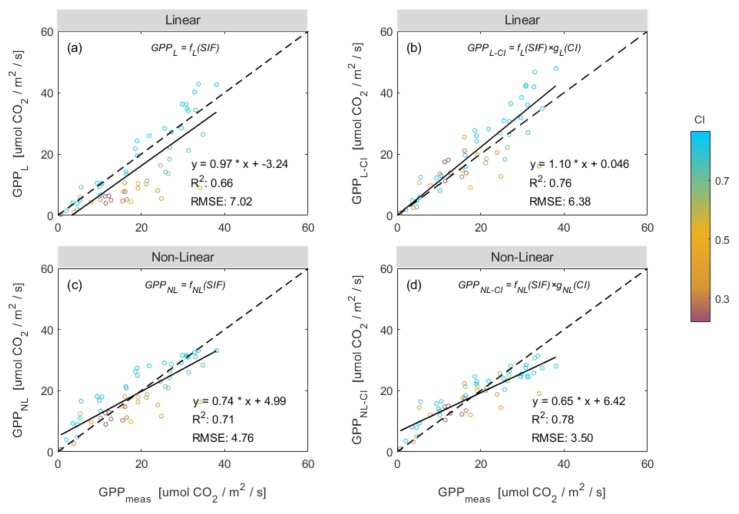
Validation of the SIF_760_-based GPP models that did and did not include the clear-sky index (CI) using the daily tower-based daily observations of the validated dataset. $GPP_{meas}$ represents the GPP measured by EC technique. $GPP_{L}$ and $GPP_{NL}$ represent the GPP predicted by the linear and non-linear model, respectively. (**a**) and (**b**) illustrate the validation results for the linear regression models, and (**c**) and (**d**) illustrate the results for the non-linear regression models. The color scale represents the CI value. The short-dashed line is the 1:1 line and the solid line represents the best-fit line.

In addition, compared to the linear model, we found the $GPP_{meas}$/$GPP_{NL}$ was not so strongly correlated with CI (Figure 8) for the non-linear SIF_760_-based GPP estimation model. Meanwhile, $GPP_{NL}$ was better related to $GPP_{meas}$ than GPP_L_, which we can see in Table 2 & Figure 9a,c. These results indicated that the non-linear SIF_760_-based GPP estimation model may partially correct the influence of CI on the SIF_760_-GPP relationship. As described in Figure 8, the correlation of $GPP_{meas}$/$GPP_{NL}$ and CI was relatively weak. However, the non-linear model could not fully correct the impact of CI, as the accuracy of GPP estimation could be further improved by integrating CI into the non-linear model (which we can see from Table 2).

In conclusion, the accuracy of GPP estimation can be improved by considering the influence of CI, especially for the linear model.

Finally, we investigated the performance of the combinations of SIF_760_ with CI in terms of GPP estimation. The SIF_760_-based GPP models with and without the inclusion of CI were trained using the 70% tower-based daily observations from 2017 and 2018, as summarized in Table 2, and the models were validated using the remaining 30% dataset. The $g\left(CI\right)$ functions were determined using the empirical statistical equations shown in Figure 7 and Figure 8, which were used to represent the impact of CI on the GPP estimation. It can be seen that, for both the linear and non-linear SIF_760_-based models, including the CI, improved the GPP estimation: the R^2^ value increased from 0.71 to 0.82 for the linear model, and from 0.82 to 0.87 for the non-linear model. These SIF_760_-based models (summarized in Table 2) were validated using the remaining 30% daily tower-based dataset, as illustrated in Figure 9. The validation results also confirmed that the SIF_760_-based GPP estimation was greatly improved by the inclusion of the CI, resulting in a higher R^2^ and a lower RMSE: the improvement was from R^2^ = 0.66 and, RMSE = 7.02 mw/m^2^/nr/sr to R^2^ = 0.76 and, RMSE = 6.38 mw/m^2^/nr/sr for the linear model, and from R^2^ = 0.71 and, RMSE = 4.76 mw/m^2^/nr/sr to R^2^ = 0.78 and, RMSE = 3.50 mw/m^2^/nr/sr for the nonlinear model. Therefore, although the SIF–GPP relationship is seriously disturbed by the meteorological conditions, this can be corrected for using the easily assessible CI metric.

## 4. Discussion

### 4.1. Possible Reasons for the Influence of CI on the SIF–GPP Relationship

In this study, we analyzed how CI affected the SIF_760_–GPP relationship for a C4 crop of maize. Our results showed that CI was an important environmental factor influencing the SIF_760_–GPP relationship, as found in previous studies [26,32,64,65,66,67]. In addition, we found that the inclusion of CI played a key role in correcting the non-linear relationship between SIF_760_ and GPP. It was observed that GPP would either saturate, or increase more slowly than SIF_760_, at high values of CI (see Figure 5 and Figure 6). There are several possible reasons for this.

First, the responses of LUE and the quantum yield of SIF (SIF_yield_) to illumination conditions are different, and CI is a sensitive indicator of illumination. In our research, we found that, for a maize field, the responses to variations in the light conditions differed between SIF_760_ and GPP. The SIF–PAR relationship showed no significant saturation phenomenon, whereas, as also observed by Damm et al. [61], for the GPP–PAR relationship, there was slight saturation at the diurnal scale (Figure 5). The light-use efficiency (LUE) is mainly influenced by the fraction of diffuse radiation. Some studies have concluded that carbon uptake is enhanced under cloudy conditions (the so-called “diffuse light fertilization effect”) [45,46], whereas the quantum yield of SIF remains relatively constant [67]. Nichol et al. [64] confirmed that the linear regression of SIF and GPP was mainly affected by the shadowed canopy fractions. Wieneke et al. [68] also found that diurnal LUE did not recover to the same level after solar soon compared to before solar soon, and that SIF_yield_ increased much more strongly than LUE. Therefore, we concluded that, CI, as a proxy for the illumination conditions, was an important factor affecting the non-linear relationships between GPP and SIF at half-hourly and daily time scales (Figure 6).

Second, CI is usually related to other environmental parameters, such as the temperature, humidity, and precipitation, which also have different influences on the LUE and SIF_yield_. Gu et al. [45] concluded that the enhanced carbon uptake under cloudy conditions might result from the interaction between various environmental factors related to the presence of clouds. Previous studies have reported that the GPP–SIF relationship is influenced by the relative humidity (RH) and air temperature. For example, Ehleringer et al. [69] reported that the quantum yield for CO_2_ uptake under normal atmospheric conditions was temperature-dependent in C3 plants but apparently not in C4 plants. Yang et al. [31] found that the ratio of GPP to SIF generally increased along with the relative humidity (RH) and confirmed that incorporating the relative humidity, the diffuse PAR fraction, and the growth stage into multiple regression analyses led to improvements in estimates of GPP for a rice paddy. In this study, although we found the VPD and AT exhibited a certain relationship with the GPP/SIF_760_, the introduction of more parameters will also accumulate, increasing the uncertainties of the model. Therefore, we only considered the influence of the CI on the relationship between SIF_760_ and GPP. In conclusion, continuous tower-based measurements can produce additional insights on how GPP and SIF react to environmental conditions and provide large amounts of reliable evidence that can be used to explore the correlation between SIF and GPP [27,29,32,35,36].

In addition, as the influence of the difference in leaf reflectance and transmittance in the far-red band becomes relatively small with an increasing interaction order [59], we ignored the effects of canopy radiative transmission on near-infrared chlorophyll fluorescence. However, as single scattering dominates in the red band, the difference in leaf reflectance and transmittance in the red band varies a lot and is not ignorable. Although red SIF indicates more information on photosynthesis, the canopy structure and varying solar-view geometries have significant effects on the red SIF compared to the far-red SIF [9,59]. Therefore, red SIF was not analyzed in this study. Future studies should pay more attention to the effects of canopy structure and its interaction with weather conditions, such as CI.

### 4.2. Limitations of the C4 Crop Experiment

This research was conducted on a routinely irrigated C4 crop. Due to differences in photosynthesis between C3 and C4 crops and differences in environmental conditions between irrigated crops and natural ecosystems, LUE and SIF_yield_ may exhibit different response patterns in other situations. The results of this study may, therefore, have limited application to other photosynthesis types and different natural stress conditions.

Several recent studies have found that the slope of the SIF–GPP relationship varies according to the biome or plant functional type (PFT) [15,19,61,70,71,72]. For example, Liu et al. [29] reported that the relationship between SIF and GPP was highly dependent on the PFT and that the photosynthesis of C3 and C4 plants exhibited different patterns in response to environmental conditions: generally, C4 plants were found to have higher photosynthetic light-use efficiencies and greater adaptation to a high light intensity than C3 plants [73,74]. Maize, a typical C4 species with a high photosynthetic capacity, usually exhibits a slight light-saturation effect at the canopy level [61,75]. This is consistent with our observations that the maize canopy GPP exhibited slight saturation for both the diurnal and daily data sets (Figure 5 and Figure 6). However, the environmental influences on C3 and C4 species are always different. Compared with C3 crops, C4 crops adapt to warm environments and have a higher light capacity for high light conditions and high temperature levels [29]. Previous studies have reported that the quantum yield of C3 plants for CO_2_ uptake, generally driven by photorespiration, decreases with an increasing temperature [69], whereas the temperature has no significant effect on the quantum yield of C4 species for CO_2_ uptake. The high evaporation demand around noon leads to a decrease in stomatal conductance and lasts longer. This effect is more pronounced in C3 leaves, leading to a further reduction in carbon absorption [6,76]. Therefore, it can be inferred that the influence of illumination on the SIF–GPP relationship for C3 plants may be greater than that for C4 plants. However, as we were limited by the data sets available in this study, our conclusion only applies to C4 plants.

In addition, as our study site consisted of irrigated farmland, there was no severe abiotic stress at the site, and our conclusions may have limited application to natural vegetation, especially vegetation under meteorological stress. Some studies have reported that the SIF–GPP relationship can be disturbed by environmental stress [12,16,21]. For example, Ač et al. [77] concluded that drought conditions might result in a decrease in steady-state red and far-red SIF. Kalaji et al. [78] reported that pea plants showed small changes in SIF parameters under cold stress. Xu et al. [79] also observed that both the canopy structure and SIF physiology changed to cope with water stress in a maize field. Therefore, in future work, more attention should be paid to other natural vegetation types as well as to exploring the specific response mechanisms of the GPP–SIF relationship to natural stress.

## 5. Conclusions

It has previously been proven that solar-induced chlorophyll fluorescence (SIF) can act as a proxy for vegetation photosynthesis. In this study, we investigated the performance of estimates of canopy GPP produced using far-red SIF and their response to different meteorological conditions by using continuous tower-based observations made in a maize field during 2017 and 2018. The SIF_760_ tracked the GPP well at both diurnal and seasonal scales, and SIF_760_ was more linearly correlated with PAR than GPP was. CI is the dominant meteorological parameter that influences the non-linear relationship between SIF_760_ and GPP, and the ratio of GPP to SIF_760_ decreased noticeably with an increasing CI at both diurnal and seasonal scales. The results showed that the SIF_760_-based GPP estimation improved from values of R^2^ = 0.66 and RMSE = 7.02 mw/m^2^/nr/sr to R^2^ = 0.76 and RMSE = 6.38 mw/m^2^/nr/sr for the linear model, and from R^2^ = 0.71 and RMSE = 4.76 mw/m^2^/nr/sr to R^2^ = 0.78 and RMSE = 3.50 mw/m^2^/nr/sr in the case of the non-linear model. These results indicate that the SIF–GPP relationship is non-linear, and that the non-linear characteristics can mostly be explained by the CI. As the CI is usually an easily accessible meteorological variable, the presented findings are useful for the remote sensing of vegetation GPP using satellite, airborne, and tower-based SIF data.

## Figures and Tables

**Table 1:** Correlation between CI, AT, and VPD with the ratio of GPP to SIF_760_ over the whole growing season in the DM maize field in 2017 and 2018.

Independent Variables	Explanatory Variables	Pearson’s Correlation Coefficient			
		2017		2018	
		30 min	Day	30 min	Day
CI	GPP/SIF_760_	0.32	0.67	0.41	0.71
VPD		0.17	0.53	0.07	0.47
AT		0.13	0.40	0.02	0.37

**Table 2:** The SIF_760_-based GPP models with and without the inclusion of the clear-sky index (CI) based on the daily tower-based observations of the training dataset. The function $g\left(CI\right)$ represents the influence of CI on the SIF_760_-based GPP models.

Regression Model	Regression Variables	Regression Equation	R^2^	RMSE
Linear	SIF	$GPP_{L}=f_{L}\left(SIF\right)=36.24\times SIF_{760}$	0.71	6.45
	SIF, CI	$GPP_{L-CI}=f_{L}\left(SIF\right)\times g_{L}\left(CI_{day}\right)$ $g_{L}\left(CI_{day}\right)=\left(-99.78\times CI_{day}+124.19\right)÷36.24$	0.82	5.71
Hyperbolic	SIF	$GPP_{NL}=f_{L}\left(SIF\right)=42.6\times \frac{SIF_{760}}{SIF_{760}+0.44}$	0.82	3.79
	SIF, CI	$GPP_{NL-CI}=f_{NL}\left(SIF\right)\times g_{NL}\left(CI_{day}\right)$ $g_{NL}\left(CI_{day}\right)=-2.16\times CI_{day}{}^{2}+1.78\times CI_{day}+0.87$	0.87	2.92
